# The effect of the JAK2 inhibitor TG101209 against T cell acute lymphoblastic leukemia (T-ALL) is mediated by inhibition of JAK-STAT signaling and activation of the crosstalk between apoptosis and autophagy signaling

**DOI:** 10.18632/oncotarget.22053

**Published:** 2017-10-23

**Authors:** Zhao Cheng, Yifang Yi, Sisi Xie, Haizhi Yu, Hongling Peng, Guangsen Zhang

**Affiliations:** ^1^ Department of Hematology, The Second Xiangya Hospital, Central South University, Changsha, Hunan, 410011, China

**Keywords:** T-ALL, apoptosis, autophagy, JAK -STAT

## Abstract

Previous reports have shown that active JAK2 contributes to T cell acute lymphoblastic leukaemia (T-ALL) development and that JAK inhibitors may be a potential treatment for T-ALL. In the current study, the JAK2 inhibitor TG101209 was used to treat T-ALL cell lines and primary T-ALL cells. The effects of TG101209 on T-ALL cells were determined, and the signaling proteins related to cell growth, apoptosis and autophagy were analysed. The results indicated that TG101209 significantly inhibited T-ALL cell proliferation and induced cell apoptosis in a dose-dependent manner. The mechanisms involved the suppression of the JAK2-STAT signaling pathway and activation of apoptosis or autophagy. Additionally, a JAK2 gene copy gain (FISH) and up-regulated JAK2, LC3 and Beclin1 expression (western blotting) were observed in T-ALL samples compared with healthy controls, which implied that JAK2 is a target for T-ALL treatment. TG101209 initiated apoptosis and autophagy in T-ALL cells; therefore, this JAK2 inhibitor may be a potential drug or alternative therapy for T-ALL.

## INTRODUCTION

T cell acute lymphoblastic leukaemia (T-ALL) is an aggressive haematopoietic malignancy that accounts for 15% of pediatric and 25% of adult ALL cases. Despite improvements in treatment over the years, approximately 25% of children and 50% of adults still fail to respond to intensive chemotherapy protocols or relapse [1]. Treatment options for T-ALL patients are limited. Standard combination chemotherapy regimens (e.g., CHOP) are relatively ineffective against T-ALL. A meta-analysis suggested that AZT therapy in combination with IFN may possess value, although morbidity and a lack of efficacy in patients with prior chemotherapy remain limitations of this regimen [2]. Antibody-based approaches, including those using daclizumab, alemtuzumab, and anti-CCR4, have shown value but are not curative [3–6]. Allogeneic bone marrow transplantation is an aggressive approach that is only curative in select patients [7]. Due to these limitations, new treatment strategies are clearly need for T-ALL.

Previous reports have demonstrated mutational activation in JAK3 in T lymphoblastic leukaemia, especially in NK/T cell lymphoma. Although the JAK2 mutation rate is low, a JAK2 fusion gene was found in T-ALL [8–11]. Last year, the Roncero AM group reported that 4 out of 16 patients with T lymphoblastic lymphoma contained a JAK2 mutation that contributed to T-ALL development. Thus, the use of JAK inhibitors may be considered for T-ALL treatment [12]. TG101209, a small-molecule Jak2-selective inhibitor, was identified by structure-based drug design and was found to be a potent inhibitor of the JAK2V617F and MPLW515L/K mutations commonly associated with primary myelofibrosis [13, 14]. To investigate the effectiveness of the JAK2 inhibitor strategy in T-ALL therapy, the current study analysed the effect of the JAK2 inhibitor TG101209 on 5 T-ALL cell lines. Flow cytometry and 3-(4, 5-dimethylthiazol-2-yl)-2,5-diphenyltetrazolium bromide (MTT) assays showed that proliferation suppression, increased apoptosis and cell cycle arrest in the G2/M phase occurred in the T-ALL cell lines with a JAK2 gene copy gain. We chose 2 of the 5 cell lines that possessed a JAK2 gene copy gain (determined by fluorescence *in situ* hybridization, FISH) and were sensitive to TG101209 in subsequent experiments. Western blotting (WB) showed that the effect of TG101209 on the cell lines with the JAK2 gene copy number alteration occurred through the JAK-STAT pathway via the regulation of the expression of JAK and STAT family proteins. Interestingly, suppression of the BCL2, Beclin1 and Light Chain 3 (LC3) proteins was also observed in the TG101209-treated T-ALL cell lines, which indicated that crosstalk between apoptosis and autophagy might also be involved in the above phenomenon. The immunostaining results were consistent with the Western blotting results. To determine whether the JAK-STAT pathway and the autophagy status correlated with T-ALL development, we collected samples from patients with T-ALL and analysed the samples by Western blotting and FISH. The results implied that the expression levels of the JAK-STAT proteins and the autophagy-related proteins Beclin1 and LC3 were up-regulated in patients with T-ALL and that most of these patients showed the JAK2 gene copy gain. Therefore, JAK2 may be a potential target for T-ALL treatment. The results from the current study indicated that the JAK2 gene copy gain and the JAK-STAT pathway were highly correlated with T-ALL development. The use of the JAK2 inhibitor TG101209 suppressed T-ALL proliferation by regulating both the JAK-STAT pathway and the crosstalk between apoptosis and autophagy and ultimately inhibiting T-ALL cell proliferation.

## RESULTS

### Patients with T-ALL showed JAK-STAT pathway activity and up-regulated autophagy

**Figure 1:**
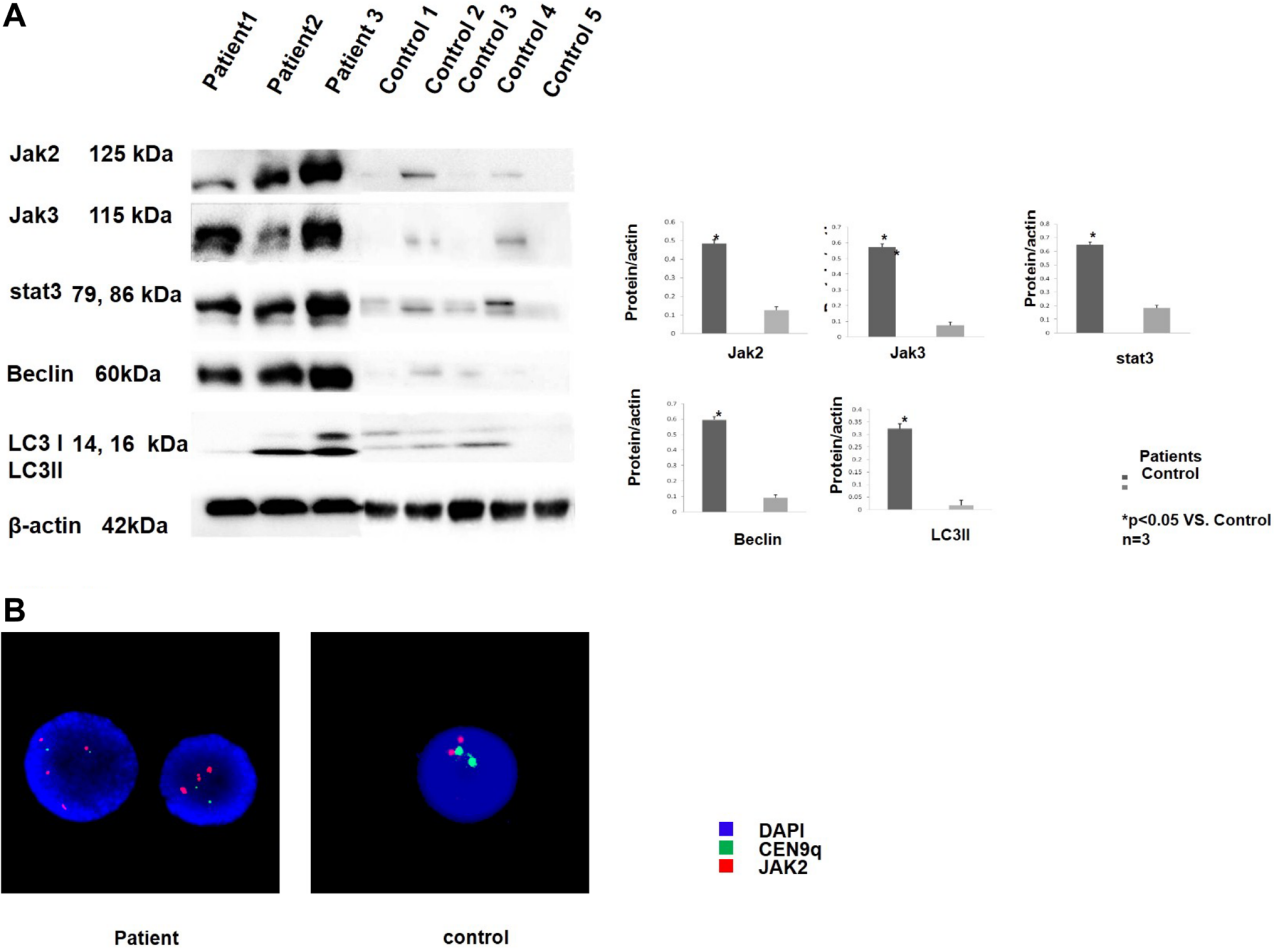
T-ALL patients showed JAK-STAT pathway activity and up-regulated autophagy (**A**) The peripheral blood mononuclear cells were collected from 3 T-ALL patients and 5 healthy controls. The cells were lysed and analysed by western blotting. An increase in the JAK-stat pathway-related proteins was observed in all 3 of the patients compared to the healthy controls as shown in the 3 upper lanes. The expression of the autophagy-related proteins was also increased in all 3 of the patients compared to the healthy control as shown in the 2 middle lanes. All of the samples were normalized to β-actin (bottom lane) Bands of western blotting were quantified by densitometry with Scion Image software (Image J 1.48u). We used the LC3B-II/loading control ratio rather than the LC3B II/LC3B-I ratio for qualifcation of LC3-II expression levels according to a newly published guideline. All the results were analysed using SPSS11.0. The graphs were listed respectively. (**B**) Representative picture of the FISH analysis. The patient samples that possessed a JAK2 copy gain (red dots) are shown on the left, and the control sample that possessed a normal JAK2 copy number (red dots) is shown on the right. All of the samples were also analysed with the CEN9q probe as an internal reference (green dots). The nuclei were all counter-stained with DAPI (blue).

The collected T-ALL patient samples were analysed by Real-time PCR and Western blotting to investigate JAK-STAT pathway activity and autophagy conditions. Comparing to normal control, the JAK/STAT pathway related genes (JAK1, JAK2, JAK3, STAT1, STAT2, STAT3 STAT5B, STAT6) were elevated in T-ALL patients (Supplementary Figure 1) The collected T-ALL patient samples were analysed by Western blotting to investigate JAK-STAT pathway activity and autophagy conditions. All patients with T-ALL showed up-regulated JAK2, JAK3, STAT3, Belclin1 and LC3 expression compared with the healthy controls; representative data are shown in Figure 1A. The JAK2 probe was applied, and the patient samples were analysed with FISH. Three patients showed a JAK2 copy gain; representative data are shown in Figure 1B. These results suggest that JAK-STAT pathway activity and autophagy may be involved in T-ALL development.

### TG101209 down-regulated the JAK-STAT pathway in T-ALL cell lines

**Figure 2:**
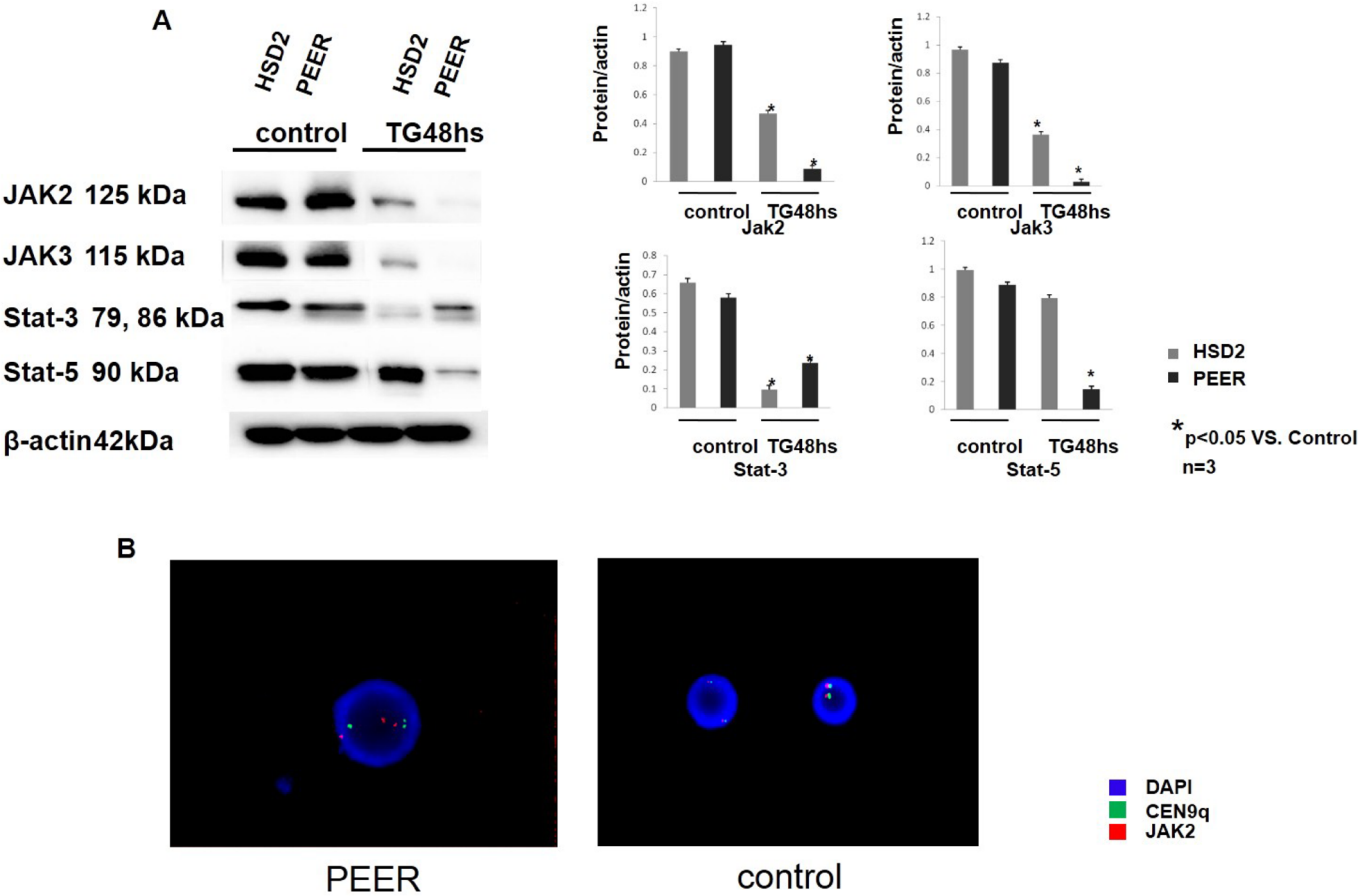
TG101209 down-regulated the JAK-STAT pathway in the T-ALL cell lines (**A**) The T-All cell lines HSD2 and PEER were treated with TG101209 for 48 hours and lysed for western blotting. Untreated cells were used as the control group. The expression levels of the JAK-stat pathway related proteins JAK2 (1st lane), JAK3 (2nd lane), Stat3 (3rd lane) and Stat5 (4th lane) were all decreased after TG101209 treatment. All of the samples were normalized with β-actin (bottom lane). Bands of western blotting were quantified by densitometry with Scion Image software (Image J 1.48u). All the results were analysed using SPSS11.0. The graphs were listed respectively. (**B**) Representative image of the FISH analysis. The PEER, which possess a JAK2 copy gain (red dots), are shown in the left. The control samples, which possesses a normal JAK2 copy number (red dots), is shown on the right. All of the samples were also analysed with the CEN9q probe as an internal reference (green dots). The nuclei were all counter-stained with DAPI (blue).

The HSD2 and PEER T-ALL cell lines were chosen for the following investigations because both of these cell lines were sensitive to TG101209. The cells were treated with TG101209 for 48 h then collected and lysed for Western blotting. The cells treated with TG101209 showed reduced JAK-STAT pathway protein expression (JAK2, JAK3, STAT3, and STAT5) compared with the control group (Figure 2A), which implied that TG101209 effectively blocked the JAK-STAT signaling pathways. To observe the JAK2 gene activity of the T-ALL cell lines, we synthesized a JAK2 probe and performed FISH analysis. PEER cells, which were the most sensitive to TG101209, showed a karyotype abnormality indicative of a JAK2 gene copy gain (Figure 2B). The JAK2 gene copy gain and the karyotype abnormality occurred at chromosome 9, which might be an important clue for the identification of TG101209-sensitive cell lines.

### TG101209 inhibits the proliferation of the T-ALL cell lines and primary bone marrow cells of T-ALL

**Figure 3:**
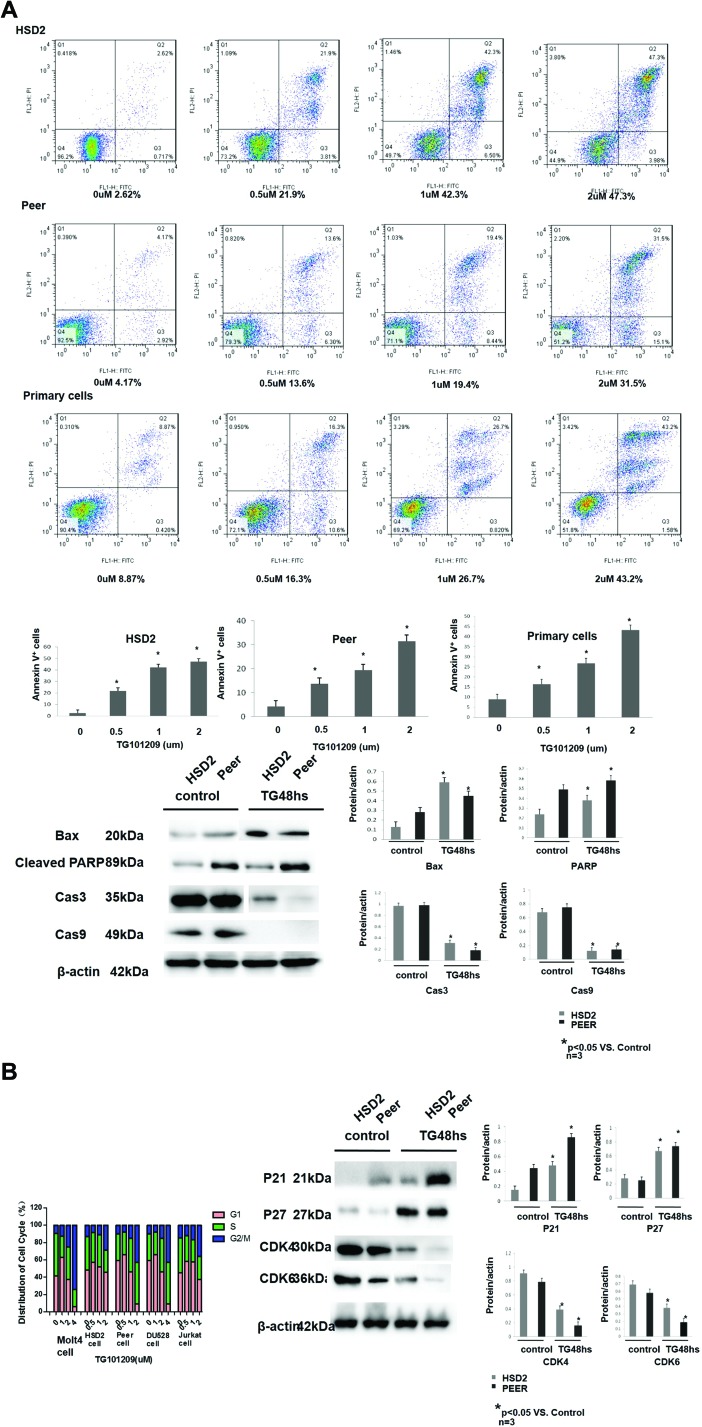
TG101209 inhibits the proliferation of the T-ALL cell lines by inducing apoptosis and cell cycle arrest (**A**) HSD2, peer and bone marrow primary T-ALLcells were treated with increasing concentrations of TG101209 for 48 h, which was followed by analysis of apoptosis by staining with PI and Annexin-V FITC. Annexin-V positive cells were measured by flow cytometry. Columns represent the average percent of Annexin V positive cells from more than 3 independent experiments, which are shown as the mean ± SD. Asterisks (^*^) represents statistically signifcant differences *p* < 0.05, respectively. Representative images are shown in the upper panel. The T-All cell lines HSD2 and PEER were treated with TG101209 for 48 hours and lysed for western blotting. Untreated cells were used as the control group. The expressions of apoptosis related proteins Bax (1st lane), CleavedPARP (2ND lane) were increased while Cas3 (3rd lane), and Cas9 (4th lane) were decreased after TG101209 treatment. All of the samples were normalized with β-actin (bottom lane). Bands of western blotting were quantified by densitometry with Scion Image software (Image J 1.48u). All the results were analysed using SPSS11.0. The graphs were listed respectively. (**B**) The Jurkat, HSD2 and PEER cell lines were treated with TG101209 (0, 0.5, 1, or 2 μM) for 48 hrs. The DU528 and MOLT-4 cell lines were treated with TG101209 (0, 1, 2, or 4 μM) for 48 hrs. The cells were collected and stained with PI, and the cell cycle distribution was analysed with flow cytometry (FACSCalibur, BD Biosciences). The data were summarized. The results showed that the cell cycle was mainly arrested in the G2/M phase in each cell line after treatment. The T-All cell lines HSD2 and PEER were treated with TG101209 for 48 hours and lysed for western blotting. Untreated cells were used as the control group. The expressions of cell cycle related proteins P21 (1st lane) , P27 (2nd lane) were increased while CDK4 (3rd lane), CDK6 (4th lane) were decreased after TG101209 treatment. All of the samples were normalized with β-actin (bottom lane). Bands of western blotting were quantified by densitometry with Scion Image software (Image J 1.48u). All the results were analysed using SPSS11.0. The graphs were listed respectively.

The DU 528, HSD2, PEER, MOLT-4 and Jurkat T-ALL cell lines were treated with TG101209 (0, 1, 2, 4, 6, 8, or 10 μM), Ruxolitib (0, 1, 2, 4, 6, 8, 10, 20 um) or Dexamethasone (1 µM, 10 µM, 100 µM or 1000 µM), and cell proliferation was analysed using MTT assay. The IC50s of the cell lines were 2.542 µM (DU528), 0.329 µM (HSD2), 0.612 µM (PEER), 2.893 µM (MOLT-4) and 1.794 µM (Jurkat) (Supplementary Figure 2). Apoptosis was increased in the HSD2 and PEER cell lines in a TG101209 concentration-dependent manner, according to the flow cytometry analysis. The expression of apoptosis-related proteins (Bax, Cleaved PARP, caspase-3 and caspase-9) was determined by Western blotting. The expression levels of Bax and Cleaved PARP were up-regulated by TG101209, while caspase-3 and caspase-9 were down-regulated in both HSD2 and PEER cell lines (Figure 3A). The cell cycle of each cell line was analysed using flow cytometry. After treatment, the cell cycle was arrested mainly at the G2/M phase. The expression of the indicated cell cycle-related proteins (P21, P27, CDK4 and CDK6) was determined by Western blotting. The expression levels of P21 and P27 were up-regulated by TG101209, while those of CDK4 and CDK6 were down-regulated by TG101209 in both the HSD2 and PEER cell lines (Figure 3B). Primary bone marrow cells from T-ALL patients and healthy controls were treated with TG101209 (0, 1, 2, 4, 6, 8, or 10 μM), and cell proliferation was analysed using MTT assay. The IC50s were 0.755um and 1.565 um respectively. (Supplementary Figure 2). Apoptosis was increased in the Primary bone marrow cells from T-ALL patients in a TG101209 concentration-dependent manner, according to the flow cytometry analysis. (Figure 3A)

### TG101209 regulates the crosstalk between apoptosis and autophagy

**Figure 4:**
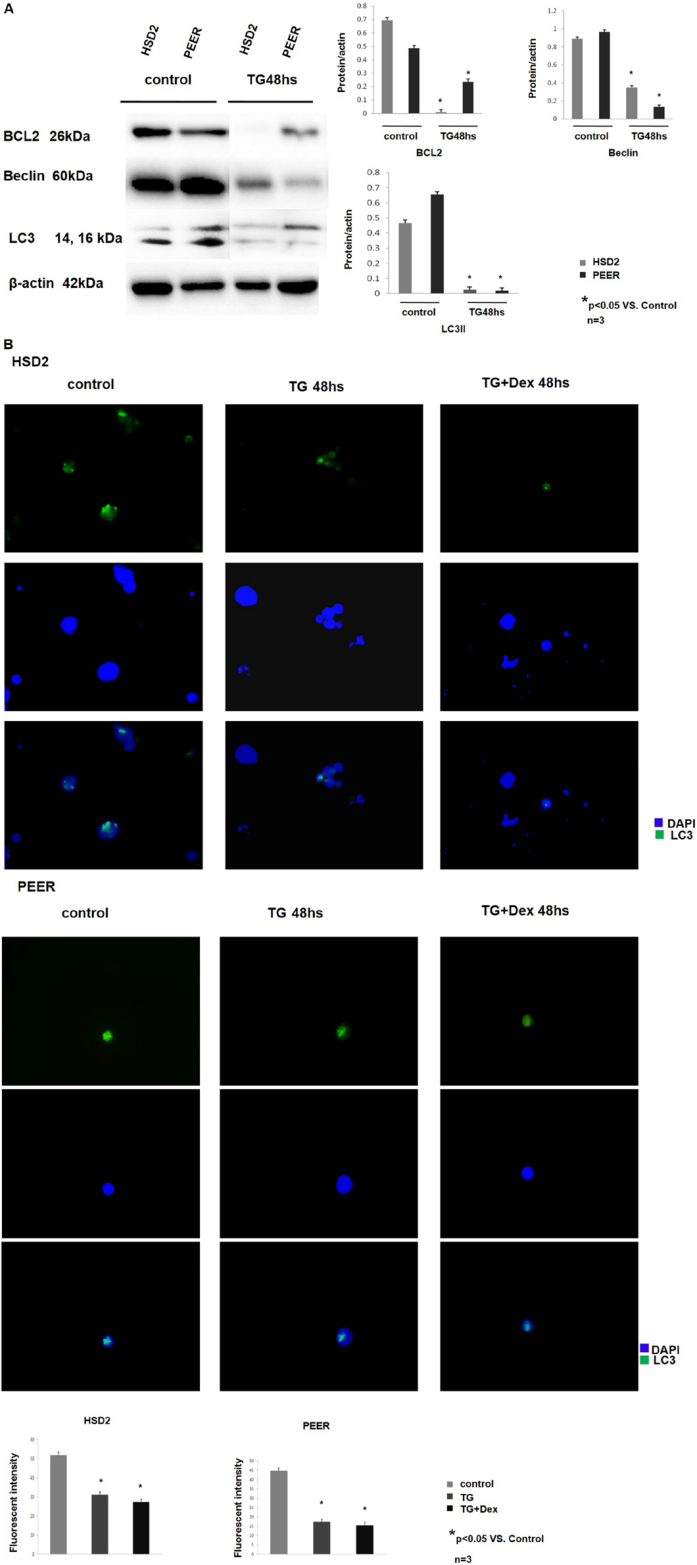
TG101209 regulates the crosstalk between apoptosis and autophagy (**A**) The T-All cell lines HSD2 and PEER were treated with TG101209 for 48 hours and lysed for western blotting. Untreated cells were used as the control group. The expression levels of BCL2 (1st lane), Beclin (2nd lane), and LC3 (3rd lane) were decreased after TG101209 treatment. All of the samples were normalized with β-actin (bottom lane). Bands of western blotting were quantified by densitometry with Scion Image software (Image J 1.48u). All the results were analysed using SPSS11.0. The graphs were listed respectively. (**B**) The HSD2 and PEER cell lines were treated with TG101209 or TG101209+Dex, and their LC3 expression levels were compared with that of the untreated group (green) by immunofluorescence cytochemistry. Representative data showed that LC3 expression was dramatically decreased after TG or TG+Dex treatment. The nuclei were all counter-stained with DAPI (blue). The fluorescence intensity of single cell was quantifed by densitometry with Scion Image software (Image J 1.48u). All the results were analysed using SPSS11.0. The graphs were listed respectively.

In addition to the JAK-STAT pathway, crosstalk between apoptosis and autophagy was also investigated using Western blotting analysis. The expression of the anti-apoptotic protein BCL2 was significantly decreased after TG101209 treatment, and the expression of the autophagy-related proteins Beclin 1 and LC3 was also decreased. These data suggest that crosstalk between apoptosis and autophagy might be involved in the TG101209-induced T-ALL cell line inhibition by the JAK/STAT/BCL2 pathway (Figure 4A). Immunofluorescence cytochemistry was performed to confirm our observations. HSD2 and PEER cells were treated with TG101209 or TG101209 + Dex, and their LC3 expression levels were compared with that of an untreated group. LC3 expression was dramatically decreased after TG or TG + Dex treatment, which was consistent with the Western blotting results (Figure 4B). The autophagy inhibition induced by TG101209 was compared with that of hydroxychloroquine sulfate (HCQ), a widely used autophagy inhibitor. We found that the effect of TG101209 (2 µM) was comparable to that HCQ (75–100 µM) in down-regulating the expression of autophagy-related proteins (BCL-2, Beclin and LC3) (Supplementary Figures 3 and 4).

## DISCUSSION

The JAK-STAT pathway plays an important role in lymphoid precursor cell proliferation, survival and differentiation [15–17]. Aberrant JAK-STAT signal transduction was first reported in T-ALLs exhibiting the t(9; 12)(p24; p13) translocation, which is a rare rearrangement resulting in a constitutively active TEL-JAK2 kinase fusion oncoprotein [18]. Activating JAK2 mutations in other haematological malignancies, such as myeloproliferative neoplasms, have demonstrated the clinical relevance of this gene and drawn more attention to its potential involvement in T-cell lymphoblastic leukaemia/lymphoma [19–21]. In a previous study, a combination of a JAK inhibitor and a Bcl-2/Bcl-xL inhibitor provided antitumour efficacy in ATL cell lines and a mouse model of human ATL [22].

Ramakrishnan’s study examined the pre-clinical activity of the JAK2 inhibitor TG101209, which induced dose- and time-dependent cytotoxicity in a variety of multiple myeloma cell lines. The induction of cytotoxicity was associated with the inhibition of cell cycle progression and the induction of apoptosis in the myeloma cell lines and patient-derived plasma cells [23]. In Pardanani’s study, TG101209 potently inhibited myeloproliferative disorder-associated JAK2V617F and MPLW515L/K mutations [13]. TG101209 was also shown to enhance radiotherapy in lung cancer models by inhibiting JAK2 signaling [24].

In the current study, the JAK2 copy gain determined by FISH and real-time PCR occurred frequently in the T-ALL cell lines and patients (Figure 1B, Figure 2B, Supplementary Figure 1), suggesting that this copy gain was the most common type of JAK2 activation in T-ALL. The efficacy of the JAK2 inhibitor TG101209 was observed in 5 T-ALL cell lines. We found that 2 out of the 5 cell lines with a JAK2 gene copy number alteration responded to the inhibitor.

Cell cycle arrest and apoptosis were induced by TG101209 through the p21/p21-CDK4/CDK6 pathway and the BAX/BCL2-caspase3/caspase9 pathway. P21 and P27, cyclin-dependent kinase (CDK) inhibitors are negative regulators of cell cycle progression. These regulators inhibit CDK activity, resulting in decreased cell proliferation [25]. After TG101209 treatment, the protein level of p21 was significantly elevated in the corresponding group compared with the control group. A similar effect on the expression level of p27, which was accompanied by a decrease in the level of CDK4 and CDK6, was also observed. The caspase and Bcl-2 protein families play crucial roles in the initiation of apoptosis [26]. Caspase-3 has been considered the major executive caspase in apoptosis. It operates as the key enzyme in the mitochondria-dependent apoptosis pathway. Therefore, measurement of caspase-3 activity is the major and the most reliable determining factor for apoptosis [27]. The Bcl-2 family of proteins include both pro-apoptotic (e.g., Bax) and anti-apoptotic (e.g., Bcl-2) members [28]. The anti-apoptotic protein Bcl-2 can regulate apoptosis through caspase-9- and -3-dependent pathways [29]. Increased Bax and decreased Bcl-2 expression can stimulate the release of cytochrome c from the mitochondria, which activates caspase-9. Caspase-9 then catalyzes the activation of caspase-3, ultimately leading to apoptosis [30]. Poly (ADP-ribose) polymerase (PARP) is a family of proteins involved in a number of cellular processes, such as DNA repair, genomic stability, and programmed cell death. PARP is inactivated by caspase cleavage. It is believed that normal inactivation occurs in systems where DNA damage is extensive. In these cases, more energy would be invested in repairing damage than is feasible; instead, that energy is retrieved for other cells in the tissue through apoptosis [31].

The expression of the anti-apoptotic protein BCL-2 and the autophagy-related protein Beclin1 was also down-regulated in addition to the JAK-STAT pathway. The BCL-2 family regulates the mitochondrial apoptosis pathway through complex interactions that determine the integrity of the outer mitochondrial membrane [32, 33]. The BCL-2 family is divided into three groups according to their functions. The first group (BCL-X, BCL-2 BCL-W and MCL-1) contains three or four BCL-2 homology (BH) domains that are necessary for the anti-apoptotic function. The second group contains BAX and BAK, which induce mitochondrial permeabilization and the release of cell death-promoting proteins. The third group consists of proteins that contain only the short BH3 domain. These BH3-only proteins interact with both anti- and pro-apoptotic BCL-2 members to induce programmed cell death [34].

In addition to participating in apoptosis, BCl-2 also plays a role in the crosstalk between apoptosis and autophagy by interacting with Beclin 1, which plays a critical role in autophagosome formation. Beclin possesses a BCL-2 homology (BH) 3 region that physically interacts with BCL-2 proteins. The binding of BCL-2 to Beclin1 reduces the capacity of Beclin 1 to activate autophagy [35, 36]. In our study, the de-regulation of both BCL-2 and Beclin 1 after TG10129 treatment implied that TG10129 might function as not only a JAK2 inhibitor to reduce the proliferation of T-ALL cells but also as a regulator of apoptosis and autophagy.

The autophagy marker LC3 was originally identified as a subunit of microtubule-associated proteins 1A and 1B (termed MAP1LC3) and was subsequently found to contain similarities to the yeast protein Apg8/Aut7/Cvt5, which is critical for autophagy. Cleavage of LC3 at the carboxy terminus immediately following synthesis yields the cytosolic LC3-I form. During autophagy, LC3-I is converted to LC3-II through lipidation by a ubiquitin-like system involving Atg7 and Atg3 that allows LC3 to become associated with autophagic vesicles. The presence of LC3 in autophagosomes and the conversion of LC3 to the lower migrating form LC3-II are used as indicators of autophagy [37, 38]. We observed that LC3 II was down-regulated in Western blotting and immunofluorescence cytochemistry assays. In a previous study, inhibiting autophagy significantly enhanced rhArg-induced cell growth inhibition and apoptosis [39]. The autophagy inhibition induced by TG101209 was compared with that of HCQ, a widely used autophagy inhibitor [40]. HCQ could down-regulate autophagy-related proteins in a concentration-dependent manner. We also showed that TG101209 (2 µM) was comparable to HCQ (75–100 µM) in terms of down-regulating the expression of autophagy-related proteins (BCL-2, Beclin and LC3) (Supplementary Figures 3 and 4). We demonstrated that TG101209 down-regulated both anti-apoptosis- and autophagy-related proteins and thus might enhance cell growth inhibition and apoptosis.

Interestingly, by analysing the T-ALL patient samples and comparing them with healthy controls, we found JAK2 gene copy gains and high expression, as well as Beclin 1 and LC3 up-regulation, which indicated that JAK2 activation plays a very important role in the development of T-ALL and that it is a target for the treatment of T-ALL. The JAK inhibitor TG101209 decreased the JAK/STAT pathway and then down-regulated BCL2, which was the key effect in the crosstalk between apoptosis and autophagy; thus, this cascade inhibited the up-regulation of autophagy, decreased the proliferation of T-ALL cells and increased their apoptosis.

Our research provide the evdence of TG101209 anti-leukemic effection *in vitro*, we need firrther clinical trial to comfirm the curitive effect, and multiple pathogenesis and different targets may limite its application, TG101209 combind with chemotherapy may work out in the future.

## MATERIALS AND METHODS

### Patient samples

The study protocol was approved by the ethics committee of the Second Xiangya Hospital, Central South University, P.R. China. Informed consent was obtained in writing in accordance with the Declaration of Helsinki.Bone marrow/peripheral blood mononuclear cells of 21 patients novel diagnosed asT-ALL (9females, 12males, median age 32) and 23 healthy controls (10 females,13males, median age 29) were collected at the second Xiangya Hospital, Central South University, P.R. China. 3 patient samples and 5 healthy control samples were used for western blotting. 5 patient samples and 5 healthy control samples were used for MTT assay. 3 patient samples and 3 heathly control samples were used for FISH. 5 patient samples and 5 healthy control samples were used for apoptosis assay. 5 patient samples and 5 healthy control samples were used for PCR. The diagnosis of T-ALL was based on morphology, cytochemistry and immunophenotyping according to the World Health Organization [41].

### T-ALL cell line maintenance

The T-ALL cell lines (HSD2, DU528, PEER, MOLT-4 and Jurkat) (Supplementary Table 1) were kindly provided by the A. Thomas Look lab at the Dana-Farber Cancer Institute at Harvard Medical School. All cell lines were maintained in RPMI 1640 medium with 10% FBS.

### MTT assay

The T-ALL cell lines (HSD2, DU528, PEER, MOLT-4 and Jurkat) were treated with various concentrations of TG101209 (Selleck, S2692) (0, 1, 2, 4, 6, 8, or 10 μM), Ruxolitib (Dacogen, Xian Janssen, Johnson-Johnsons, USA) (0,1,2,4,6,8,10,20μM) and Dexamethasone (D4902, Sigma-Aldrich, Merck) (0,1,10,100,500,1000 μM) respectively for 48 h. Bone marrow cells collected from patients with T-ALL were treated with various concentrations of TG101209 (0, 1, 2, 4, 6, 8, or 10 μM) for 48h. MTT assay was conducted following the instructions provided by the manufacturer. The IC50 value was determined for each cell line. All experiments were repeated 3 times.

### Flow-cytometry (cell cycle)

Jurkat, HSD2 and PEER cells were treated with TG101209 (0, 0.5, 1, or 2 μM) for 48 h. DU528 and MOLT-4 cells were treated with TG101209 (0, 1, 2, or 4 μM) for 48 h. The cells were collected and stained with propidium iodide (PI). The cell cycle distribution was determined using flow cytometry (FACSCalibur, BD Biosciences). All experiments were repeated 3 times.

### Flow cytometry (apoptosis)

HSD2 cells , Peer cells and primary bone marrow cells derived from T-ALL patients were treated with TG101209 (0, 0.5, 1, or 2 μM) for 48 h respectively. Cells were double stained with Annexin V-FITC /PI and analysed by flow cytometry to determine the apoptosis status (FACSCalibur, BD Biosciences). All experiments were repeated at least 3 times.

### Western blotting

The Western blotting analysis was performed as previously described [42]. Briefly, the cells were treated with TG101209 (2 µM) or hydroxychloroquine sulfate (HCQ) (Selleck, S4430) (25, 50, 75, 100 µM) for 48 h or were collected from the clinical T-ALL patients. The cells were harvested, and an equivalent amount of the cell lysate was subjected to sodium dodecyl sulfate polyacrylamide gel electrophoresis (SDS-PAGE) and then transferred to polyvinylidene fluoride (PVDF) membranes for the Westsern blotting assay. The membranes were incubated with the JAK2 (D2E12) XP^®^ Rabbit monoclonal antibody (mAb) (Cell Signaling Technology, #3230), STAT3 (124H6) mouse mAb (Cell Signaling Technology, #9139), STAT5 antibody (Cell Signaling Technology, #9363), Bcl-2 (50E3) rabbit mAb (Cell Signaling Technology, #2870), Bax (D2E11) rabbit mAb (Cell Signaling Technology, #5023S ) , PARP (46D11) Rabbit mAb (Cell Signaling Technology, #9532S), caspase-3 (8G10) Rabbit mAb (Cell Signaling Technology, #9665S) and ,caspase-9 (D2D4) Rabbit mAb, #7237S Beclin-1 antibody (Cell Signaling Technology, #3738), and LC3 (Cell Signaling Technology, #12741) antibody and the appropriate horseradish peroxidase (HRP)-linked anti-rabbit/mouse IgG secondary antibodies (Cell Signaling Technology, #7074), according to the species of the primary antibody. The membranes were treated with an enhanced chemiluminescent detection kit (Thermo Fisher Scientific, #17097), and the signals were collected and photographed (Bio-Rad, Gel Doc, XR). All experiments were repeated 3 times. These bands were quantified by densitometry with Scion Image software (ImageJ 1.48u). We used the LC3B-II/loading control ratio rather than the LC3B II/LC3B-I ratio to quantify LC3-II expression levels according to a newly published guideline [43].

### Immunofluorescence cytochemistry

The Peer and HSD-2 cell lines were treated with TG101209 (2 µM), HCQ (50 µM, 100 µM) or TG101209 (1 µM) + Dex (100 µM) for 48 h and then collected and fixed in ice-cold 100% methanol for 15 min at -20°C. Then, the cells were smeared onto an amino silane (APS)-coated slide. After air dying, the samples were blocked in blocking buffer (1X PBS, 5% normal serum, and 0.3% Triton™ X-100) for 60 min at room temperature. LC3 was diluted in antibody dilution buffer (1X PBS, 1% bovine serum albumin (BSA), and 0.3% Triton X-100) at a 1:100 ratio and incubated at 4°C overnight, followed by incubation with a fluorochrome-conjugated secondary antibody diluted in antibody dilution buffer for 1 h at room temperature in the dark. The slides were also incubated with the ProLong Gold Antifade reagent with DAPI (Cell Signaling Technology, #8961). The samples were observed and imaged using a Zeiss AX10 Scope A1. All experiments were repeated 3 times. The fluorescence intensity of single cell was quantified by densitometry with Scion Image software (ImageJ 1.48u).

### Fluorescence *in situ* hybridization (FISH)

The JAK2/CEN9q FISH probe (Cat No. FA0225, Lot No. GC158-GBCBI) was purchased from the ABNOVA (Taiwan) Corporation. The size of the JAK2 probe was approximately 290 kb, the fluorophore was Texas red, and the location was 9q24. The size of the CEN9q probe was approximately 470 kb, the fluorophore was FITC, and the location was 9p21. FISH was performed following the manufacturer’s instructions. Cell lines treated with TG101209 or patient samples were seeded and treated with colchicine (20 µg/ml); then, the cells were centrifuged, resuspended in hypotonic solution and incubated in a 37°C water bath for 1 h. The cells were fixed with fixing solution (methanol:triacetate = 3:1). After dehydration with 95% EtOH, the cells were smeared onto a slide, and the FISH probe was applied. The samples were denatured at 75°C for 5 min, and hybridization was performed in a humidified box at 37°C for 24 h. After hybridization, the samples were dipped into 2X SSC at room temperature for 5 min, followed by 2X SSC/0.3% NP-40 at 75°C for 2 min, and then finally transferred into 2X SSC and incubated at room temperature for 1 min. DAPI was applied as a counter-stain, and the target area was covered with a glass cover slip. The samples were observed and imaged using an Olympus BX50 fluorescence microscope. All experiments were repeated 3 times.

## CONCLUSIONS

Based on the above evidence, JAK2 inhibitor -TG101209 may be an alternative choose for the therapy of T-ALL in the near future.

## SUPPLEMENTARY MATERIALS FIGURES AND TABLE



## Abbreviations

T-ALL: T cell acute lymphoblastic leukemia
MTT: 3-(4,5-dimethylthiazol-2-yl)-2,5-diphenyltetrazolium bromide assay
FISH: fluorescence *in situ* hybridization
EtOH: ethanol CH3CH2OH
Dex: Dexamethasone, DXMS
TG: TG101209
