# Supplementary material for: The effect of the JAK2 inhibitor TG101209 against T cell acute lymphoblastic leukemia (T-ALL) is mediated by inhibition of JAK-STAT signaling and activation of the crosstalk between apoptosis and autophagy signaling
Source: Oncotarget. 2017 Oct 23;8(63):106753–63. doi: 10.18632/oncotarget.22053 (PMC5739771; doi:10.18632/oncotarget.22053)
Supplement: Supplementary file 1 [file oncotarget-08-106753-s001.pdf]

The effect of the JAK2 inhibitor TG101209 against T cell acute lymphoblastic leukemia (T-ALL) is mediated by inhibition of JAK-STAT signalling and activation of the crosstalk between apoptosis and autophagy signalling

SUPPLEMENTARY MATERIALS

Supplementary Table 1: Cell lines information

Cell Line	MYC level	p-AKT ser473	Notch1	PTEN	FBXW7	CDKN2A	Oncogene Group	GSI treatment
Jurkat	W	S	wt	del	mut	del	TAL1	resistant
Peer	S	S	ND		-	del	other	ND
MOLT4	S	W	HD and PEST	mut	-	no-del, mut	TAL1	resistant
DU528	M	M	wt	-	mut	no-del	TAL1	resistant
HSD2	-	-	wt	-	mut	-	TAL1	resistant

The T-ALL cell lines (HSD2, DU528, PEER, MOLT-4 and Jurkat) were kindly provided by the A. Thomas Look lab at the Dana-Farber Cancer Institute at Harvard Medical School. The relative informations, MYC expression level, the phosphrylated AKT at ser 47,mutations of Notch, PTEN, FBXW7, CDKN2A genes, oncogene group and GSI treatment reaction were listed in the table above. “W” represent weak expression, “S” represent strong expression, “M” represent medium expression. “mut” represent mutation, “del” represent deletion.

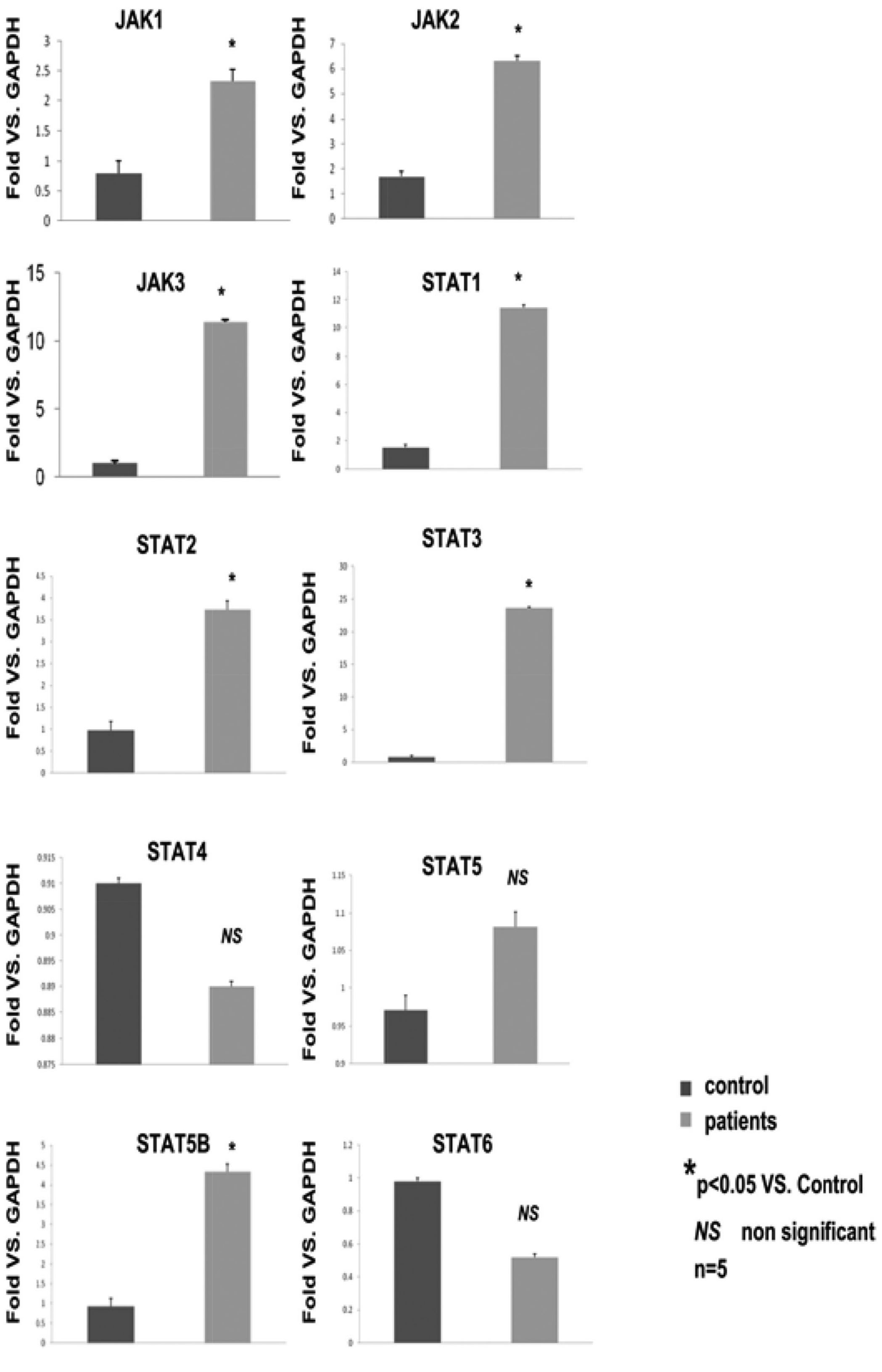

Supplementary Figure 1: Bone marrow mononuclear cells were collected from 5 T-ALL patients and 5 healthy control, the RNA were extracted and reverse transcript to cDNA, then real-time PCR was performed to determine the JAK/STAT pathway related genes (JAK1,JAK2,JAK3, STAT1,STAT2, STAT3, STAT4, STAT5A, STAT5B, STAT6). Columns represent the fold increase normalized to GAPDH of each genes from more than 5independent experiments, which are shown as the mean ± SD. Results showed that comparing to normal control, the JAK/STAT pathway related genes (JAK1, JAK2, JAK3, STAT1, STAT2, STAT3 STAT5B, STAT6) were elevated in T-ALL patients.

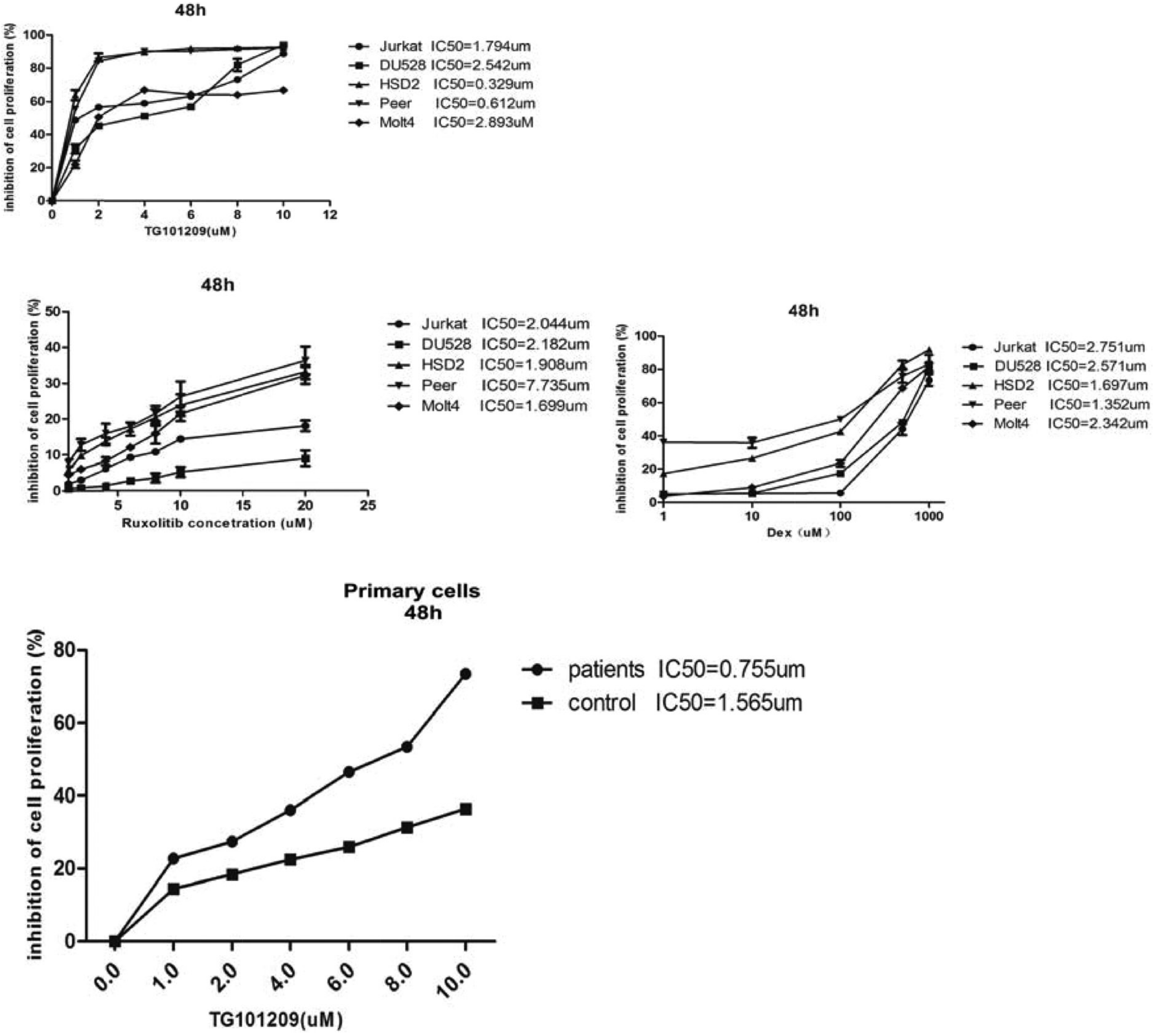

Supplementary Figure 2: The T-ALL cell lines DU 528, HSD2, PEER, MOLT-4 and Jurkat were treated with TG101209, Ruxolitib and Dex respectively, shown on the X axis , and analysed using MTT assay. The results were listed out respectively. The primary bone marrow cells derived from T-ALL patients were treated with TG101209 and analyzed using MTT assay. The results were shown on the bottom.

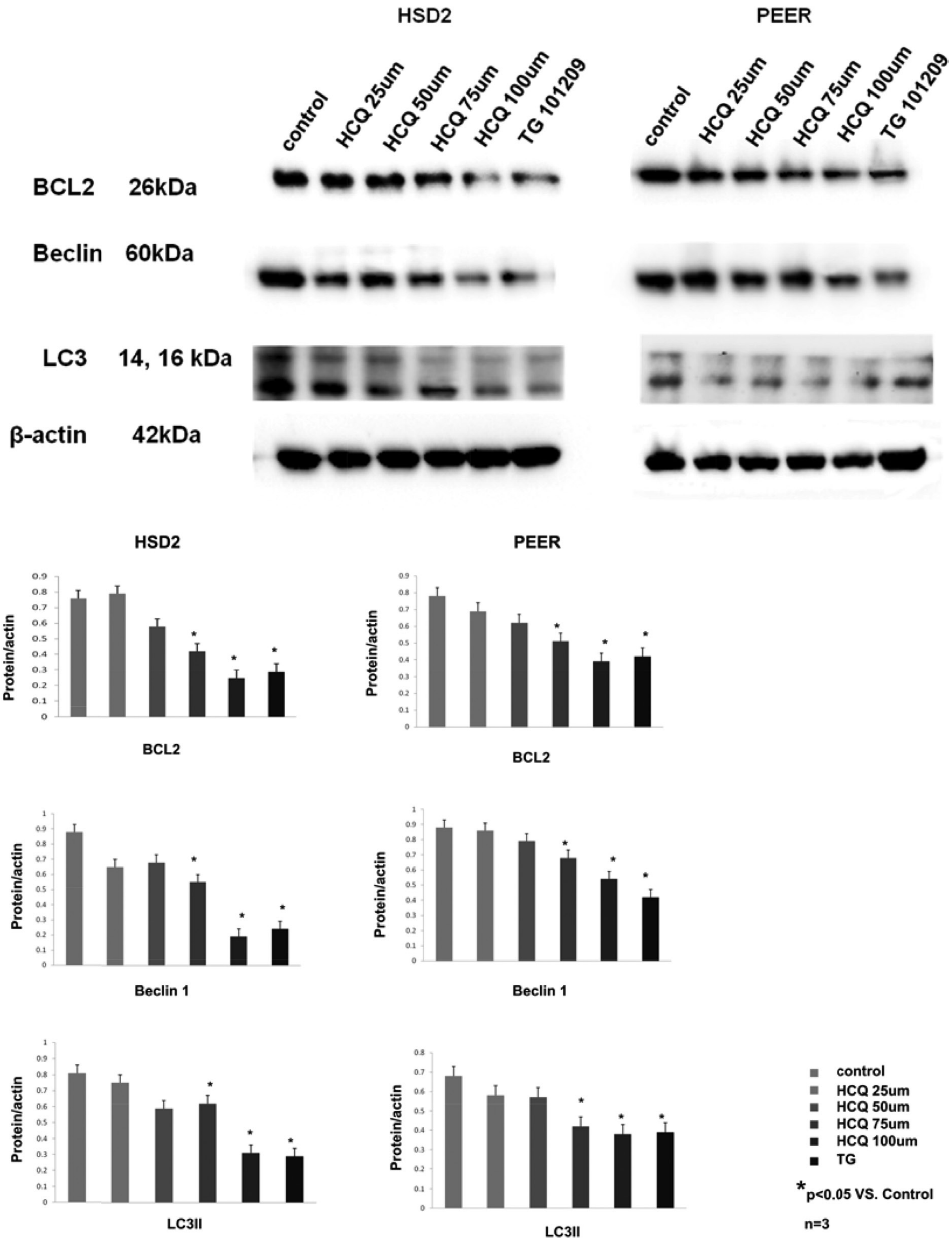

Supplementary Figure 3: The T-All cell lines HSD2 and PEER were treated with HCQ (25 um, 50 um, 75 um , 100 um) TG101209 (2 um)for 48 hours respectively and lysed for western blotting. Untreated cells were used as the control group. The expressions of autophagy related proteins BCL2 (1st lane), Beclin (2nd lane) and LC3II (3rd lane) were all decreased after HCQ (in a concentration dependent way) and TG101209 treatment. All of the samples were normalized with β-actin (bottom lane). Bands of western blotting were quantified by densitometry with Scion Image software (Image J 1.48 u). All the results were analysed using SPSS11.0. The graphs were listed respectively.

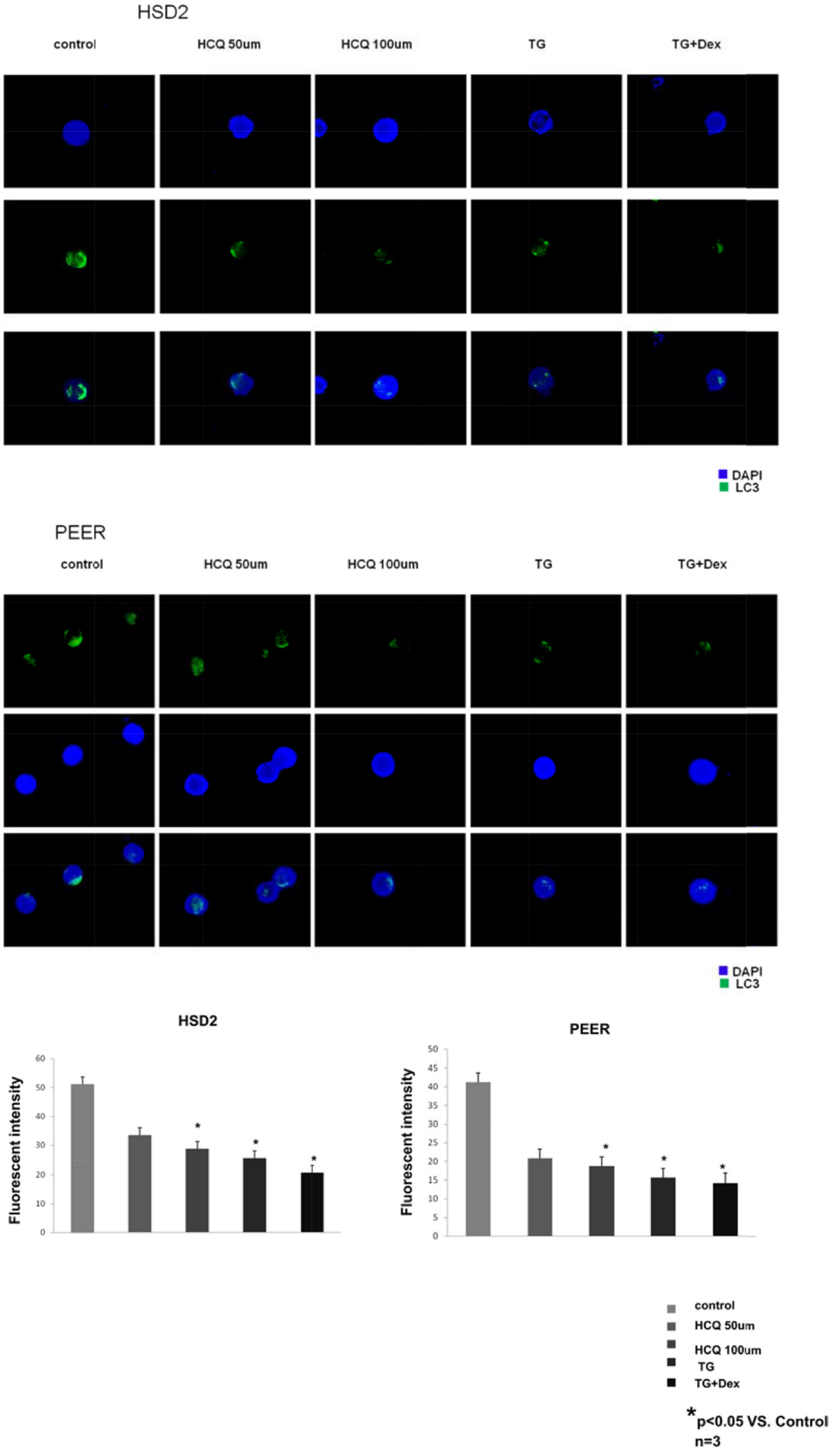

Supplementary Figure 3: The HSD2 and PEER cell lines were treated with HCQ (50 um, 100 um), TG101209 (2 um)or TG101209 (1 um) + Dex (100 um) respectively, and their LC3 expression levels were compared with that of the untreated group (green) by immunofluorescence cytochemistry. Representative data showed that LC3 expression was dramatically decreased after TG or TG + Dex treatment which are comparable with HCQ (100 um) treatment. The nuclei were all counter-stained with DAPI (blue). The fluorescence intensity of single cell was quantifed by densitometry with Scion Image software (Image J 1.48 u). All the results were analysed using SPSS11.0. The graphs were listed respectively.
